# A higher prognostic nutritional index is inversely associated with the need for renal replacement therapy in elderly critically Ill surgical patients

**DOI:** 10.1186/s12893-025-03240-w

**Published:** 2025-10-21

**Authors:** Qi Liu, Simin Li, Hailin He, Qiufeng Liao, Rongxing Bao, Xiaolin Gu, Dandong Luo, Chongjian Zhang

**Affiliations:** Department of Cardiac Surgical Intensive Care Unit, Guangdong Provincial People’s Hospital (Guangdong Academy of Medical Sciences), Southern Medical University, No. 106, Zhongshan 2Nd Road, Guangzhou City, Guangdong Province 510080 People’s Republic of China

**Keywords:** Prognostic Nutritional Index, Renal Replacement Therapy, Acute Kidney Injury, Surgical ICU, Elderly Patients

## Abstract

**Background:**

Acute kidney injury requiring renal replacement therapy (RRT) is a critical complication in elderly surgical patients in the intensive care unit (ICU) and is associated with high mortality and healthcare costs. The prognostic nutritional index (PNI), calculated as 10 × serum albumin level (g/dL) + 0.005 × total lymphocyte count (per mm^3^), integrates both the serum albumin level and lymphocyte count to reflect a patient's nutritional and immunological status, however, its association with the need for RRT remains underexplored. This study aimed to evaluate the association between the PNI and need for RRT in critically ill surgical patients aged ≥ 65 years.

**Methods:**

A secondary analysis of 3,406 elderly surgical patients in the ICU (2015–2020) from a single-center cohort was conducted. The PNI was calculated serum albumin levels and lymphocyte counts obtained at ICU admission. Patients were stratified into PNI tertiles (low: 26.50–41.00; middle: 41.50–48.50; high: 49.00–73.00). Multivariate logistic regression and subgroup analyses were applied to explore the association of the PNI with the need for RRT. Furthermore, we also examined the association between the PNI and the need for RRT by employing restricted cubic splines. The discriminative ability of the PNI was assessed using receiver operating characteristic (ROC) curves and the area under the curve (AUC).

**Results:**

According to the multivariate regression models, the PNI demonstrated a significant inverse association with the need for RRT after comprehensive covariate adjustment. The adjusted odds ratio (OR) for the need for RRT was 0.95 (95% CI 0.93–0.97; *P* < 0.0001) per 10-unit increase in the PNI. When the PNI was analyzed by tertile, patients in the middle (OR = 0.72, 95% CI: 0.54–0.97, *P* = 0.0285) and high tertiles (OR = 0.42, 95% CI: 0.29–0.60, *P* < 0.0001) presented a progressively lower risk of RRT than did those in the low tertile, with a significant dose‒response trend (*P* for trend < 0.0001). We further performed exploratory subgroup analyses and confirmed that higher PNI levels were independently associated with a lower risk of RRT (*P* for interaction > 0.05). The RCS analysis suggested a linear relationship between the PNI and the need for RRT (*P* for nonlinearity = 0.2848). The E-value of 2.59 demonstrates the robustness of the results against unmeasured confounding.

**Conclusion:**

In geriatric surgical patients (≥ 65 years) presenting with critical illness, an elevated PNI demonstrated an inverse correlation with the need for RRT.

**Supplementary Information:**

The online version contains supplementary material available at 10.1186/s12893-025-03240-w.

## Introduction

Critically ill surgical patients face substantial clinical challenges due to postoperative complications, including acute kidney injury (AKI), which frequently necessitates renal replacement therapy (RRT) and is associated with increased mortality and healthcare costs. AKI occurs in 20–40% of surgical patients admitted to the intensive care unit (ICU), with RRT needed in approximately 5–10% of patients. Notably, patients requiring RRT have a mortality rate exceeding 50%, underscoring the urgency of identifying modifiable risk factors and early predictors for timely intervention [1, 2].

Current guidelines for initiating RRT rely on traditional biomarkers such as the serum creatinine concentration and urine output, which often involve delayed measurements or are confounded by nonrenal factors [3]. This diagnostic lag underscores the need for novel biomarkers that are capable of identifying high-risk patients before irreversible renal damage occurs. Mechanistically, nutritional status may reflect a maladaptive "malnutrition‒inflammation‒immunodeficiency" axis that exacerbates AKI progression by impairing tissue repair and amplifying inflammatory kidney injury [4].

Nutritional status is a crucial factor for the survival of patients receiving RRT. The nutritional requirements of these patients are increased due to the considerable loss of water-soluble electrolytes, glucose, amino acids, and vitamins as well as increases in systemic inflammation, protein catabolism, and heat loss during RRT [5]. Many nutritional factors, including low body mass index, low initial serum albumin levels, low protein intake, and carnitine deficiency, have been associated with increased mortality in patients receiving RRT [6, 7].

The prognostic nutritional index (PNI), developed by Buzby [8] and later modified by Onodera and Kosaki [9], is a readily accessible marker for evaluating nutritional and inflammatory status. This index has been linked to postoperative or peri-treatment morbidity and mortality across various patient groups, including those with various malignancies, heart failure and diabetes mellitus (DM) [10, 11]. Research has revealed that the PNI is a prognostic factor for outcomes or AKI risk assessment in individuals with critical illness, including acute coronary syndrome, major abdominal surgery, ICU admission and coronavirus disease 2019 (COVID-19) [12–14]. Kurtul et al. demonstrated that a PNI of less than 38 had 82% sensitivity and 70% specificity for predicting contrast-associated AKI in patients with ST-segment elevation myocardial infarction who underwent percutaneous coronary intervention [12]. Shimoyama reported that a PNI with a cutoff point of 26.08 had 58% sensitivity and 67% specificity for predicting sepsis-associated AKI in enrolled ICU patients with sepsis [15]. However, studies have predominantly focused on mortality or infection endpoints, leaving a critical knowledge gap regarding the association of AKI with organ-specific interventions such as RRT.

Elderly surgical populations exhibit unique risk profiles due to heterogeneous surgical stressors and baseline comorbidities, necessitating targeted investigations [16]. On the basis of the available evidence, this study examined the relationship between the PNI and the need for RRT in elderly critically ill surgical patients and is the first evidence-based exploration in this specific population. This study aimed to evaluate the association of the PNI with the need for RRT in elderly critically ill surgical patients through a secondary analysis.

## Methods

### Study population and design

This study constitutes a secondary retrospective analysis, indicating that we performed additional analyses of data from an existing dataset rather than collecting new data specifically for this research. We obtained the dataset from a previously published study [17]. The dataset is available through the website (10.1371/journal.pone.0304627 IF:2.6Q2B3), which was originally shared by Duc Trieu Ho et al. We have cited the dataset as follows: 10.1371/journal.pone.0304627.s006 IF: NA NA NA. The data used in this study can be downloaded for free from the public data repository, which contains raw data uploaded by various authors to render their research data discoverable, freely reusable, and referable.

**Fig. 1:**
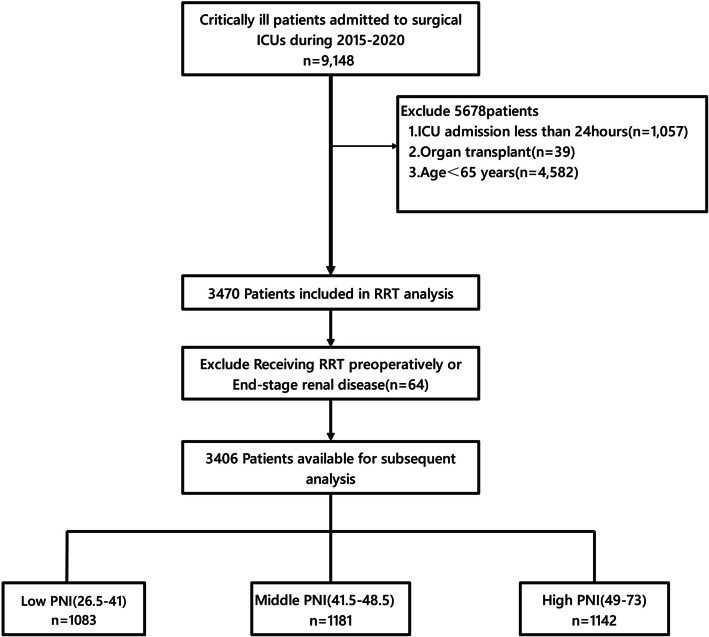
Flowchart of patient enrollment

The original investigation was a retrospective, observational cohort study. This study was conducted at Taichung Veterans General Hospital (TCVGH), a referral hospital with three surgical intensive care units (SICUs) in central Taiwan, and included consecutive critically ill patients admitted to the SICUs between 2015 and 2020. The exclusion criteria for the original research were as follows: patients admitted to the ICU for less than 24 h and organ transplant recipients. Among the 9,148 patients initially assessed for study eligibility, 8,052 met the criteria for inclusion. The present study constitutes a post hoc analysis of the data derived from the original research. We excluded patients aged less than 65 years, patients who received preoperative RRT, and those with end-stage renal disease to evaluate the effect of the PNI on the risk of RRT in elderly patients (Fig. 1). Notably, new ethics permissions and consent to participate were not applicable, given that the original author obtained ethical approval during the initial research. Our study represents a retrospective analysis of previously collected data.

### Data collection

The database includes data on the following variables collected at ICU admission: demographic characteristics, the Charlson Comorbidity Index (CCI), the Acute Physiology and Chronic Health Evaluation (APACHE) II score, laboratory data, surgical information, and the receipt of RRT during ICU stay. Our analysis was based on a complete dataset with no missing values. The PNI was calculated on the basis of serum albumin levels and lymphocyte counts obtained at ICU admission.

### Grouping and outcome definitions

All participants were categorized into three groups on the basis of PNI tertile. The PNI was calculated using the following formula:

10 × serum albumin level (g/dL) + 0.005 × total lymphocyte count (/mm^3^) [18]. The thresholds for each tertile were defined as follows: Tertile 1 (T1, *n* = 1083): PNIs between 26.50 and 41.00; Tertile 2 (T2, *n* = 1181): PNIs between 41.50 and 48.50; and Tertile 3 (T3, *n* = 1142): PNIs between 49.00 and 73.00.

The study endpoints were focused on the occurrence of RRT events following surgical intervention. The decision regarding the timing and indications for RRT initiation was guided by the clinical assessment and judgment of the local multidisciplinary care team.

### Statistical analysis

Continuous variables are expressed as the mean ± standard deviation (normal distribution) or median (quartile) (skewed distribution), and categorical variables are expressed as the frequency or percentage. One-way analysis of variance (ANOVA) (normal distribution) and the chi-square test (categorical variables) were used to test for differences among the different PNI groups. The regression coefficient and corresponding 95% confidence intervals (CIs) were calculated using unadjusted and multivariate-adjusted logistic regression analyses to determine associations between the PNI and the need for RRT. The PNI was modeled both continuously and categorically on the basis of tertiles. On the basis of established evidence and previously published literature, covariates were selected using a directed acyclic graph (DAG) (Supplementary Fig. 1). Potential multicollinearity was evaluated by calculating the variance inflation factor (VIF), and variables with VIF > 5 were excluded from the final multivariate logistic regression model [19] (Supplementary Table 1). Model 1 was adjusted for no variables. Model 2 was adjusted for sex, age, body mass index, and the Charlson comorbidity index, and Model 3 was adjusted for the covariates in Model 2 plus additional covariates, including surgical division, emergency surgery status, scheduled surgery status, APACHE II score, platelet count, creatinine level, and hemoglobin level. We performed stratified analyses and tested for interactions across clinically relevant subgroups to assess the robustness of our findings. Furthermore, we also examined the associations between PNI tertile and the study endpoints by employing restricted cubic splines.The discriminative ability of the CCI,CCI plus PNI,APACHEⅡ,APACHEⅡ plus PNI was assessed using receiver operating characteristic (ROC) curves and the area under the curve (AUC).

The data were analyzed using Empower (R) (www.empowerstats.com, X&Y solutions, Inc., Boston MA) and R software (version 4.3; R Foundation, http://www.R-project.org/). A two-tailed *P* value < 0.05 was considered to indicate statistical significance in all analyses.

## Results

### Characteristics of the included patients

A total of 3,406 adults were included in the final analysis (Fig. 1). The need for RRT rate was 9% (*n* = 308). Patients were divided into tertiles according to the PNI: T1: PNIs between 26.50 and 41.00 (*n* = 1083); T2: PNIs between 41.50 and 48.50 (*n* = 1181); and T3: PNIs between 49.00 and 73.00 (*n* = 1142). The baseline characteristics of the study patients by PNI tertile are presented in Table 1.

**Table 1:** Baseline characteristics

PNI tertile	Low (26.50–41.00)	Middle (41.50–48.50)	High (49.00–73.00)	*P* value
N	1083	1181	1142	
Demographics and comorbidities				
Age, years	76.00 (70.00–83.00)	75.00 (69.00–81.00)	73.00 (68.00–79.00)	< 0.001
Sex, number (%)				< 0.001
Female	386 (35.64%)	461 (39.03%)	512 (44.83%)	
Male	697 (64.36%)	720 (60.97%)	630 (55.17%)	
Body mass index (kg/m^2^)	23.00 (21.00–26.00)	24.00 (21.00–26.00)	24.00 (22.00–27.00)	< 0.001
Charlson comorbidity index	2.00 (1.00–3.00)	2.00 (1.00–3.00)	2.00 (1.00–3.00)	< 0.001
Laboratory data				
Platelet count (10^3^/μL)	280.90 (238.90–335.90)	273.90 (232.90–329.90)	273.90 (236.90–326.90)	0.191
Creatinine level (mg/dL)	1.10 (0.80–1.60)	1.00 (0.80–1.40)	1.00 (0.80–1.30)	< 0.001
Hemoglobin level (g/dL)	12.50 (11.70–13.60)	12.90 (11.90–14.00)	13.20 (12.20–14.30)	< 0.001
Absolute lymphocyte count (10^3^/μL)	1.30 (0.90–1.60)	1.90 (1.40–2.40)	3.10 (2.40–4.00)	< 0.001
Albumin level (g/dL)	3.10 (2.90–3.30)	3.50 (3.30–3.70)	3.90 (3.60–4.20)	< 0.001
White blood cell count (10^3^/μL)	13.70 (10.90–17.00)	14.10 (11.30–16.90)	14.90 (12.40–17.50)	< 0.001
Surgical division				< 0.001
Neurosurgical division	363 (33.52%)	553 (46.82%)	588 (51.49%)	
Cardiovascular surgical division	185 (17.08%)	251 (21.25%)	297 (26.01%)	
General-colorectal surgery divisions	314 (28.99%)	218 (18.46%)	137 (12.00%)	
Other surgical division	221 (20.41%)	159 (13.46%)	120 (10.51%)	
Type of surgery				
Emergency surgery				0.046
No	862 (79.59%)	958 (81.12%)	955 (83.63%)	
Yes	221 (20.41%)	223 (18.88%)	187 (16.37%)	
Scheduled surgery				< 0.001
No	608 (56.14%)	611 (51.74%)	504 (44.13%)	
Yes	475 (43.86%)	570 (48.26%)	638 (55.87%)	
Severity and managements				
APACHE II score	23.00 (18.00–27.00)	22.00 (16.00–26.00)	21.00 (16.00–25.00)	< 0.001
Shock				< 0.001
No	886 (81.81%)	1051 (88.99%)	1044 (91.42%)	
Yes	197 (18.19%)	130 (11.01%)	98 (8.58%)	
Mechanical ventilation				< 0.001
No	427 (39.43%)	539 (45.64%)	622 (54.47%)	
Yes	656 (60.57%)	642 (54.36%)	520 (45.53%)	
Outcomes				
RRT				< 0.001
No	929 (85.78%)	1080 (91.45%)	1089 (95.36%)	
Yes	154 (14.22%)	101 (8.55%)	53 (4.64%)	

Compared with patients with low PNIs, patients with high PNIs were younger and had lower APACHE II scores, a lower incidence of mechanical ventilation, lower Charlson comorbidity indices, a lower incidence of shock, and a lower incidence of RRT. There was a significant difference in the surgical division, type of surgery, creatinine level, hemoglobin level, absolute lymphocyte count, albumin level, and white blood cell count between the groups (*P* < 0.05). There was no significant difference in the platelet count between the groups (*P* > 0.05) (as shown in Table 1).

### Univariate analysis of factors associated with RRT in elderly surgical patients in the ICU

The results of the univariate analysis of factors associated with the risk of RRT are presented in Table 2. Age, sex and platelet count were not significantly associated with the need for RRT. However, a greater body mass index, a greater Charlson comorbidity index, a greater APACHE II score, the presence of shock, the need for mechanical ventilation, general-colorectal surgery division status, other surgical division status and the creatinine level were positively associated with the study endpoints. In contrast, the hemoglobin level and neurosurgical division were inversely associated with the study endpoints. Additionally, the analysis of the PNI and its tertiles demonstrated that higher PNIs were significantly associated with a reduced risk of RRT.

**Table 2:** Univariate analysis of factors associated with RRT in elderly surgical ICU patients

Variables	Statistics	OR (95%CI)	*P* value
Age, years	75.52 ± 7.70	1.00 (0.99, 1.02)	0.7693
Sex			
Female	1359 (39.90%)	1	
Male	2047 (60.10%)	0.85 (0.67, 1.08)	0.1757
Body mass index (kg/m^2^)	23.92 ± 4.24	1.05 (1.03, 1.08)	< 0.0001
Charlson comorbidity index	2.04 ± 1.58	1.44 (1.35, 1.54)	< 0.0001
Platelet count (10^3^/μL)	284.00 ± 58.58	1.00 (1.00, 1.00)	0.2428
Creatinine level (mg/dL)	1.13 ± 0.41	3.58 (2.70, 4.74)	< 0.0001
Hemoglobin level (g/dL)	13.08 ± 1.47	0.78 (0.71, 0.85)	< 0.0001
Surgical division			
Neurosurgical division	1504 (44.16%)	1	
Cardiovascular surgical division	733 (21.52%)	2.65 (1.87, 3.76)	< 0.0001
General-colorectal surgery divisions	669 (19.64%)	4.04 (2.90, 5.63)	< 0.0001
Other surgical divisions	500 (14.68%)	3.91(2.74, 5.59)	< 0.0001
Emergency surgery			
No	2775 (81.47%)	1	
Yes	631 (18.53%)	1.25 (0.94, 1.66)	0.127
Scheduled surgery			
No	1723 (50.59%)	1	
Yes	1683 (49.41%)	0.97 (0.77, 1.23)	0.7934
APACHE II score	21.55 ± 6.84	1.09 (1.07, 1.11)	< 0.0001
Shock			
No	2981 (87.52%)	1	
Yes	425 (12.48%)	1.60 (1.17, 2.19)	0.003
Mechanical ventilation			
No	1588 (46.62%)	1	
Yes	1818 (53.38%)	2.23 (1.73, 2.88)	< 0.0001
PNI	45.85 ± 8.07	0.93 (0.92, 0.95)	< 0.0001
PNI tertile			
Low	1083 (31.80%)	1	
Middle	1181 (34.67%)	0.56 (0.43, 0.74)	< 0.0001
High	1142 (33.53%)	0.29 (0.21, 0.41)	< 0.0001

### Multivariate regression analysis of the effect of the PNI on the risk of RRT

The results of the multivariate regression analysis evaluating the association between the PNI and the risk of RRT in elderly surgical ICU patients are presented in Table 3. As a continuous variable, in Model 1, each 10-unit increase in PNI was associated with a 50% reduction in the odds of RRT (OR = 0.50, 95% CI: 0.43–0.60, *P* < 0.0001). In Model 2, each 10-unit increase in PNI remained significantly associated with a 51% reduction in RRT risk (adjusted OR = 0.49, 95% CI: 0.41–0.58; *P* < 0.0001).In Model 3, even after extensive adjustments,each 10-unit increase in PNI decreased the odds of RRT by 40% (OR = 0.60, 95% CI 0.50–0.72, *P* < 0.0001).While patients with PNI > 42.75 had 53% lower odds compared to those with PNI ≤ 42.75 (OR = 0.47, 95% CI 0.36–0.61, *P* < 0.0001). When stratified by tertile, patients in both the middle tertile (OR = 0.72; 95%CI: 0.54–0.96; *P* = 0.0285) and high tertile (OR = 0.42; 95%CI: 0.29–0.60; *P* < 0.0001) presented progressively reduced risks of RRT relative to those in the low tertile, with a significant dose‒response relationship (*P* for trend < 0.0001).These findings suggest that higher PNIs are independently associated with a reduced risk of RRT, even after accounting for a wide range of clinical and surgical confounders.

**Table 3:** Multivariate regression analysis of the effect of the PNI on the risk of RRT

Exposure	Model 1		Model 2		Model 3	
	OR (95%CI)	*P* value	OR (95%CI)	*P* value	OR (95%CI)	*P* value
PNI per 10u	0.50 (0.43,0.60)	< 0.0001	0.49 (0.41,0.58)	< 0.0001	0.60 (0.50, 0.72)	< 0.0001
PNI dichotomous						
≤ 42.75	1		1		1	
> 42.75	0.36 (0.28,0.46)	< 0.0001	0.36(0.28,0.46)	< 0.0001	0.47 (0.36,0.61)	< 0.0001
PNI tertile						
Low	1		1		1	
Middle	0.56 (0.43,0.74)	< 0.0001	0.57(0.44,0.75)	< 0.0001	0.72(0.54,0.97)	0.0285
High	0.29 (0.21,0.41)	< 0.0001	0.30(0.21,0.41)	< 0.0001	0.42(0.29,0.60)	< 0.0001
*P* for trend		< 0.0001		< 0.0001		< 0.0001

Model 1: adjusted for no variables

Model 2: adjusted for sex, age, body mass index, and Charlson comorbidity index

Model 3: adjusted for sex, age, body mass index, Charlson comorbidity index, surgical division, emergency surgery status, scheduled surgery status, APACHE II score, platelet count, creatinine level, and hemoglobin level

### Subgroup analysis examining the association between the PNI and the risk of RRT

Subgroup analysis was performed to evaluate the potential associations between the PNI and the risk of RRT among elderly surgical ICU patients, stratified according to various clinical and demographic factors. When analyzed as a continuous variable, the PNI generally suggested a reduced risk of RRT across all predefined subgroups—including sex, surgical division, emergency surgery, scheduled surgery, and mechanical ventilation. All interaction *P*-values exceeded 0.05, which may indicate a consistent relationship between PNI and RRT risk throughout these subgroups. Detailed results are presented in Table 4. Overall, these findings suggest that the PNI may be associated with a lower risk of RRT across diverse patient populations.

**Table 4:** Subgroup analysis examining the association between the PNI and the risk of RRT

Variables	N	OR (95%CI)	*P* value	*P* for interaction
Sex				0.1546
Female	1359	0.94 (0.91, 0.96)	< 0.0001	
Male	2047	0.95 (0.93, 0.97)	< 0.0001	
Surgical division				0.0782
Neurosurgical division	1504	0.93 (0.89, 0.97)	0.0003	
Cardiovascular surgical division	733	0.93 (0.90, 0.97)	0.0003	
General-colorectal surgery divisions	669	0.97 (0.94, 1.01)	0.0982	
Other surgical division	500	0.97 (0.93, 1.00)	0.0709	
Emergency surgery				0.9115
No	2775	0.95 (0.93, 0.97)	< 0.0001	
Yes	631	0.95 (0.91, 0.99)	0.0097	
Scheduled surgery				0.0824
No	1723	0.96 (0.93, 0.98)	0.0004	
Yes	1683	0.94 (0.91, 0.96)	< 0.0001	
Mechanical ventilation				0.6362
No	1588	0.95 (0.92, 0.98)	0.0004	
Yes	1818	0.95 (0.92, 0.97)	< 0.0001	

### AUC of CCI, CCI + PNI, APACHE II, APACHE II + PNI for predicting RRT risk

**Fig. 2:**
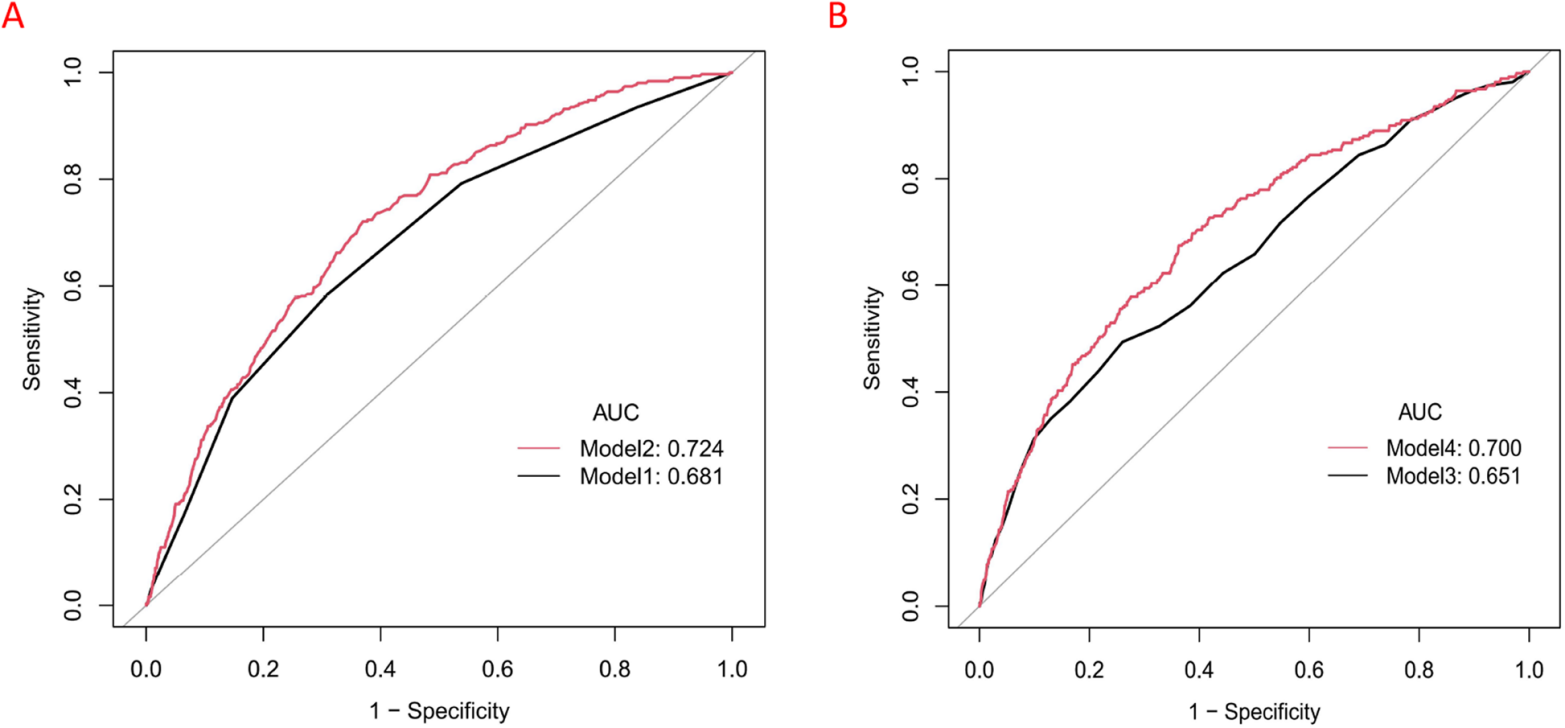
ROC analysis of the PNI (**A**) model 1 + model 2, (**B**) model 3 + model 4, Model 1: CCI,Model 2: CCI + PNI,Model 3: APACHE II,Model 4: APACHE II + PNI

The ROC curve was plotted to measure the predictive power of CCI, CCI + PNI, APACHE II, APACHE II + PNI. Table 5 and Fig. 2 shows the predicted values of PNI.ROC analysis revealed a moderate discriminative ability of the PNI for predicting the need for RRT, with an AUC of 0.647 (95% CI 0.616–0.678). The optimal cutoff value, determined by the Youden index, was 42.75, corresponding to a sensitivity of 61.7% and a specificity of 63.2%.The integration of the PNI with established risk scores significantly enhanced prognostic discrimination. When added to the CCI, PNI increased the AUC from 0.681 (95% CI: 0.650–0.712) (Model 1, Fig. 2A) to 0.724 (95% CI: 0.696–0.753)(Model 2, Fig. 2A), representing an absolute improvement of 0.043 (*P* < 0.001).Similarly, PNI supplementation to APACHE II elevated the AUC from 0.651 (95% CI: 0.617–0.684)(Model 3, Fig. 2B) to 0.700 (95% CI: 0.668–0.731)(Model 4, Fig. 2B), with a delta of 0.049 (*P* < 0.001).These improvements surpassed the minimal clinically important difference of 0.02 for AUC, highlighting PNI’s ability to capture malnutrition-related risk pathways that are not adequately represented by comorbidity burden (CCI) or acute physiological disturbances (APACHE II) alone.

**Table 5:** AUC of CCI, CCI + PNI, APACHE II, APACHE II + PNI for predicting RRT risk

Model	AUC	95%CI	*P* value
Model 1: CCI	0.681	0.650–0.712	0.0002
Model 2: CCI + PNI	0.724	0.696–0.753	
Model 3: APACHE II	0.651	0.617–0.684	< 0.0001
Model 4: APACHE II + PNI	0.700	0.668–0.731	

*Abbreviations: CCI* Charlson comorbidity index, *APACHE II* Acute physiology and chronic health evaluation. *PNI* Prognostic nutritional index

The results indicate that integrating PNI with conventional risk assessment tools—particularly through the CCI + PNI or APACHE II + PNI combinations—provides valuable complementary prognostic insight, demonstrating significant clinical utility.

### Relationship between the PNI and the need for RRT according to the restricted cubic spline analysis

**Fig. 3:**
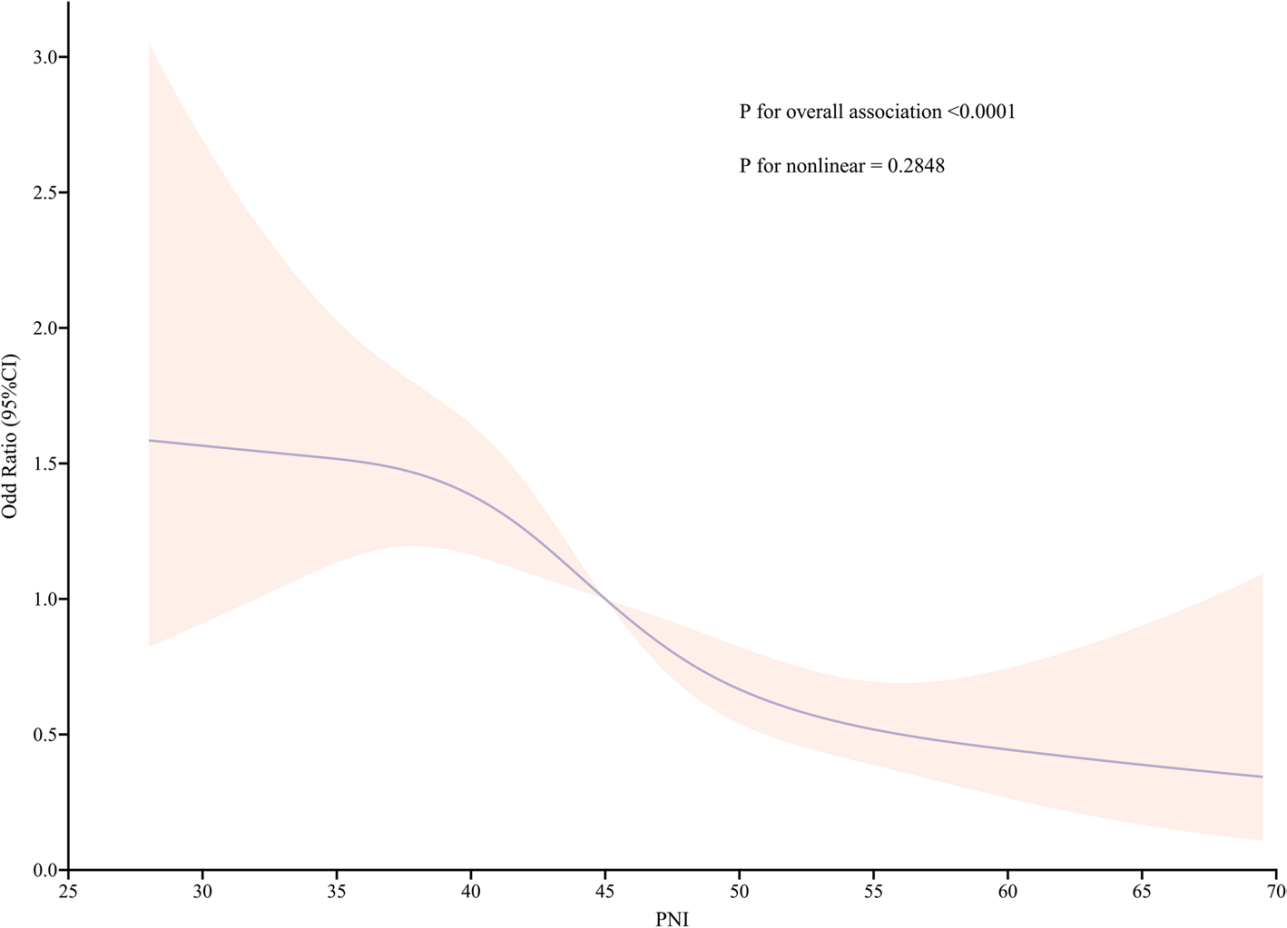
Restricted cubic spline demonstrating the association between the PNI and the need for RRT

Restricted cubic spline analysis demonstrated an inverse, linear association between the PNI and the risk of RRT (*P* for nonlinearity = 0.2848). Across the entire cohort, each incremental increase in the PNI was associated with a progressive decrease in the risk of RRT (Fig. 3).

### Sensitivity analysis

Supplementary Table 2 presents sensitivity analyses that examine the association between the PNI and RRT risk in ICU patients. The results strongly support our original conclusions: PNI is a highly significant protective factor against RRT after adjusting for multiple covariates such as age, sex, comorbidities, and disease severity, every 10-unit increase in PNI was associated with an OR of 0.54 (95% CI: 0.45–0.66, *p* < 0.0001) for the outcome "RRT" compared to "no event."PNI also serves as a protective factor against mortality (OR = 0.73, *P* < 0.0001), confirming that death is indeed a competing event associated with PNI.It is noteworthy that the strength of the association between PNI and RRT (OR = 0.54) is even greater than its association with mortality (OR = 0.73). This finding robustly demonstrates that even though the competing risk of death may have diluted the true effect of PNI on RRT by "removing" some high-risk patients with low PNI, we still detected a highly significant and strong negative association between PNI and RRT. This indirectly suggests that, in the absence of competing risks, this association would likely be even stronger.

Additionally, the calculated E-value of 2.59 (Supplementary Fig. 2) suggests stability and indicates that unmeasured confounders are unlikely to substantially affect the results.

## Discussion

Our study revealed a significant inverse association between the PNI and the risk of RRT in elderly critically ill surgical patients. In this secondary analysis of a retrospective, observational cohort study, lower PNIs were independently and strongly associated with the need for RRT. Restricted cubic splines demonstrated a continuous, inverse, and linear relationship between the PNI and the risk of RRT (*P* for nonlinearity = 0.28).The integration of the PNI with established risk scores significantly enhanced prognostic discrimination.The addition of the PNI significantly improved the predictive accuracy of two established clinical risk scores. When combined with the CCI, the model's performance (AUC) increased by an absolute 0.043. Similarly, adding PNI to the APACHE II score resulted in an even greater improvement, with an AUC increase of 0.049. Both enhancements were statistically significant (*P* < 0.001) and exceeded the minimal clinically important difference. This demonstrates that PNI provides substantial incremental value by capturing malnutrition-related risks that are not fully measured by comorbidity or acute physiological scores alone. These associations remained consistent across clinically relevant subgroups. To our knowledge, this study represents the first investigation of the association between the PNI and the need for RRT in an elderly critically ill population. These findings highlight the potential of the PNI as a valuable tool for early risk stratification and intervention in elderly surgical patients in the ICU.The E-value of 2.59 demonstrates the robustness of the results against unmeasured confounding.

Nutrition is essential for the maintenance of life. Malnutrition results from inadequate consumption of macro- and micronutrients, and the American Society for Parenteral and Enteral Nutrition (ASPEN) defines malnutrition as the presence of 2 or more of the following: insufficient energy intake, localized or generalized fluid retention, muscle loss, subcutaneous fat mass and/or weight loss, and impaired functional status [20]. In older individuals, malnutrition is particularly common in those requiring ICU stays, those staying in nursing homes and those in palliative services for a long time because these individuals are unable to maintain normal feeding processes due to reduced appetite, severe illness, and unconsciousness, especially in the first week of critical illness [21, 22]. A multinational retrospective study demonstrated that only a small proportion (14%) of hospitalized older patients had normal nutritional status [23]. Patients in the ICU have varying degrees of malnutrition, with a prevalence ranging from 30 to 50%. [Assessment of nutritional status in the critically ill]. Malnutrition is a complex problem that is still largely unacknowledged. According to reports, malnutrition impacts 20–50% of acute care patients, with greater rates in intensive care patients [24]. Chronic and acute starvation, as well as the intensity of the underlying pathophysiological conditions that cause ICU admission, impact the nutritional status of ICU patients.

The PNI comprises an assessment of albumin, a major component of plasma protein, and lymphocytes, which are important cells in immunity and indicate a patient’s nutritional and immune status [25]. The PNI, as a tool to assess the probability of RRT in ICU patients, has advantages. The PNI is easy to acquire because albumin and lymphocyte counts are routinely measured by blood biochemistry examinations upon ICU admission, and there is no additional cost. This dual physiological derangement, characterized by hypoalbuminemia-driven fluid imbalance and lymphopenia-mediated systemic inflammation, may exacerbate renal functional compromise, thereby increasing susceptibility to dialysis-dependent organ failure [26, 27]. Malnutrition, characterized by hypoalbuminemia and lymphopenia, compromises tissue repair and amplifies systemic inflammation, creating a maladaptive malnutrition‒inflammation‒immunodeficiency axis that exacerbates AKI progression. Hypoalbuminemia, a key component of the PNI, is linked to increased vascular permeability, oxidative stress, and impaired antioxidant capacity, which collectively aggravate renal tubular injury and interstitial inflammation [28].Concurrently, lymphopenia, which is reflective of cellular immune dysfunction, diminishes the ability of the body to mitigate infection-driven inflammatory cascades, further perpetuating renal damage [29]. This synergy between nutritional depletion and immune suppression fosters a proinflammatory milieu, marked by elevated levels of cytokines such as interleukin-6 and tumor necrosis factor-alpha, which are known to impair renal microcirculation and promote apoptosis in renal tubular cells [30]. Moreover, malnutrition impedes adaptive responses to surgical stress, including inadequate protein synthesis for tissue repair and weakened antioxidant defenses against ischemia‒reperfusion injury, both of which are critical in AKI pathogenesis [31]. Studies have demonstrated that a low PNI is correlated with increased levels of inflammatory markers and reduced levels of anti-inflammatory mediators, creating a feedback loop that accelerates renal functional decline [32]. These mechanisms align with our findings, where lower PNI tertiles were associated with higher rates of shock and mechanical ventilation. By integrating nutritional and immunological metrics, the PNI provides a holistic risk stratification tool, highlighting actionable pathways for early interventions such as immunonutrition or anti-inflammatory therapies to attenuate AKI progression and reduce the need for RRT in high-risk surgical cohorts.

Bacterial translocation and systemic inflammatory responses directly impact albumin levels and lymphocyte counts, which are critical components of the PNI. Given that both hypoalbuminemia and lymphopenia are independently linked to adverse clinical outcomes [33], the prognostic utility of the PNI likely stems from the integrated contributions of its two components. The increased permeability of capillaries, release of cytokines, and hepatic reprioritization of protein synthesis during systemic inflammation lead to a decrease in circulating albumin levels [34]. Hypoalbuminemia subsequently disrupts oncotic pressure, contributing to fluid shifts, tissue edema, and impaired perfusion, which ultimately compromise organ function and healing [35]. The lymphocyte count, another key element of the PNI, is suppressed by both malnutrition and the systemic inflammatory environment [36].

Many studies have reported that a low PNI is significantly associated with postoperative complications, including surgical site infection, leakage, bleeding, cardiopulmonary failure, and pulmonary embolism [37, 38].Albumin is commonly employed to evaluate malnutrition, particularly in patients with chronic kidney disease who are undergoing dialysis.Previous studies have highlighted the importance of the PNI across various diseases. For example, Dolapoglu et al. and Hu et al. reported a significant correlation between the PNI and the incidence of AKI in patients undergoing coronary procedures [39, 40], and the PNI was shown to be an independent predictor of delirium in elderly patients after spinal surgery, hip fracture surgery, total hip arthroplasty, and noncardiac surgery [41, 42].

Optimal screening for malnutrition is performed in the first 24–48 h of hospital admission [43]. Our study extends these findings to the unique population of patients receiving RRT, who have both a high nutritional demand and increased systemic inflammation. The study by Yu-Fu Lee et al. focused on the associations between the PNI and RRT-free survival as well as mortality in critically ill patients [26], whereas our study emphasized the relationship between the PNI and the need for RRT in elderly critically ill surgical patients. These findings demonstrate that the PNI holds clinical significance both before and after RRT initiation.

Additionally, Jia-Jin Chen et al. demonstrated that the PNI could effectively serve as a risk stratification tool for identifying individuals at low risk for AKI in populations with a relatively low incidence of AKI. The diagnostic accuracy of their assessment remained consistent across various AKI criteria and two mean serum albumin level groups. The sensitivity appears to be greater in medical patients than in surgical patients (0.72 vs. 0.55), potentially reflecting a higher baseline postoperative inflammatory status in surgical patients than in medical patients. We concluded that, compared with these novel biomarkers, the PNI is routinely available without additional cost in daily clinical practice, making it a practical and cost-effective option for AKI risk assessment [44].

However, as Ginga Suzuki noted, recent studies have demonstrated that the optimal timing for RRT initiation remains uncertain [45]. An international consensus conference in intensive care medicine indicated that hyperkalemia, metabolic acidosis, and pulmonary edema unresponsive to diuretic therapy are recognized criteria for initiating RRT [46, 47]. In the study by Stéphane Gaudry’s team, RRT initiation was deferred until the appearance of life-threatening indications (refractory hyperkalemia, metabolic acidosis, or pulmonary edema) or until blood urea nitrogen (BUN) concentrations increased to 140 mg/dL [48]. Ginga Suzuki et al. reported that nonemergency RRT initiation was determined by either a BUN level ≥ 112 mg/dL or oliguria (< 500 mL/day for > 72 h). The secondary outcomes included the RRT initiation rate, ICU length of stay, and 60- and 90-day mortality rates. Notably, the authors emphasized that the use of specific BUN thresholds as a standalone criterion for RRT initiation remains controversial. While the BUN level alone is unlikely to be a reliable indicator, its combination with oliguria may provide a more clinically reasonable approach [45]. Mikko J. et al. developed novel predictive models to determine the optimal timing for ICU admission and continuous renal replacement therapy (CRRT) initiation. These findings demonstrate that these models, which incorporate comprehensive laboratory, clinical, and demographic data collected at both ICU admission and CRRT initiation, have significant predictive value [49]. The management of RRT (including the choice of the intermittent or continuous technique, the duration and interval between sessions, the device setting, and the anticoagulation modality) was left to the discretion of each study site and was prescribed and monitored according to national guidelines [50]. This selection of different modalities of RRT for patients in critical care is natural, as CRRT is considered the modality of choice for hemodynamically unstable patients with the most severe illness [49]. The STARRT-AKI Investigators recommended that clinicians delay initiating RRT unless one or more of the following criteria are met: (1) hyperkalemia (serum potassium level ≥ 6.0 mmol/L), (2) severe metabolic acidosis (pH ≤ 7.20 or serum bicarbonate level ≤ 12 mmol/L), (3) severe respiratory failure (PaO₂/FiO₂ ratio ≤ 200) with clinical signs of volume overload, or (4) persistent AKI lasting ≥ 72 h after randomization [51].

Therefore, the available literature indicates significant variability in the indications for RRT, with no universally accepted clinical criteria established across different institutions. Other retrospective studies using RRT as an endpoint similarly failed to provide explicit initiation criteria.

This analysis revealed significant associations between a composite evaluation of nutritional and immunological status derived from serum albumin and lymphocyte measurements and the need for RRT initiation in an elderly surgical ICU population [52]. By quantifying the interplay between protein-energy malnutrition and immune system dysregulation, this metric reflects systemic vulnerability linked to escalating renal compromise necessitating dialysis [53]. These findings highlight the clinical importance of evaluating nutritional and immunological derangements when assessing renal vulnerability, particularly in critically ill patients, where systemic physiological stressors may accelerate renal functional decline necessitating RRT. By bridging the gap between nutritional status and organ-specific outcomes, this study supports a paradigm shift toward proactive, nutrition-focused care in elderly surgical ICU patients.

Incorporating the PNI into routine assessment at ICU admission rapidly identifies surgical patients with concurrent nutritional and immune compromise who are at increased risk for adverse outcomes, enabling prompt, individualized interventions and informed resource allocation. Systematic PNI integration can guide personalized, evidence-based care pathways that increase long-term survival and enhance recovery, addressing a critical gap in risk stratification for this vulnerable cohort.

We acknowledge that there are several limitations to this study.We acknowledge several limitations in this study. First, as a secondary analysis, our findings are inherently limited by the original study’s design, potentially introducing selection bias. Although we adjusted for measurable confounders, unmeasured variables and residual bias due to the retrospective design may persist. All subgroup analyses were exploratory and not corrected for multiple comparisons. Thus, results require cautious interpretation, and prospective studies with predefined criteria are warranted. Additionally, although PNI was laboratory-based, we could not control for variations in standardization, sampling timing, or unmeasured confounders. Differences in techniques and reference ranges may also introduce bias.Second, despite confounding adjustment, unmeasured factors such as variations in RRT initiation or undocumented renal function may contribute to residual confounding. The lack of granular temporal data on ICU mortality prevented competing risk analysis, potentially leading to conservative effect estimates if early death limited RRT opportunity.Third, PNI cutoff values lack standardization across studies. As a nutritional marker, PNI does not assess body composition or micronutrients. A single measurement at ICU admission cannot reflect dynamic nutritional or immune changes—particularly relevant in AKI, where albumin and lymphocyte levels vary. Absence of serial measurements restricted analysis of temporal trends or prognostic utility over time.Fourth, results from this single-center Asian cohort may not be generalizable. Although the nutrition-inflammation–RRT risk pathway is likely broadly relevant, our PNI thresholds and effect estimates require external validation in diverse populations and settings.Fifth, RRT indications vary widely across institutions, with no universally accepted standards. As with other retrospective studies using RRT as an endpoint, we could not specify precise initiation criteria, modalities, or timing.Sixth, future studies should prospectively evaluate whether PNI-guided care improves outcomes and reduces avoidable RRT. Quantifying PNI’s added value to standard metrics would strengthen evidence and support clinical translation.Finally, this study focused on short-term in-hospital outcomes. The relationship between PNI and long-term renal function remains unknown. Longitudinal studies should assess whether PNI-based stratification can guide post-discharge care,such as enhanced nephrology follow-up and monitoring,to mitigate chronic kidney disease progression.

## Conclusion

This study confirmed a significant association between the PNI and the risk of RRT in elderly surgical patients in the ICU, with lower PNI values being associated with a greater need for RRT. As a simple and readily available marker of nutritional and immune status, the PNI has important clinical value.

## Supplementary Information

Supplementary Material 1

Supplementary Material 2

Supplementary Material 3

Supplementary Material 4

## Data Availability

This study constitutes a secondary retrospective analysis, indicating that we performed additional analyses of data from an existing dataset rather than collecting new data specifically for this research. We obtained the dataset from a previously published study [17]. The dataset is available through the website (10.1371/journal.pone.0304627 IF: 2.6 Q2 B3), which was originally shared by Duc Trieu Ho et al. We have cited the dataset as follows: 10.1371/journal.pone.0304627.s006 IF:NANANA. The data used in this study can be downloaded for free from the public data repository, which contains raw data uploaded by various authors to render their research data discoverable, freely reusable, and referable.

## Abbreviations

RRT: Renal replacement therapy
ICU: Intensive care unit
PNI: Prognostic nutritional index
AKI: Acute kidney injury
SICUs: Surgical intensive care units
TCVGH: Taichung Veterans General Hospital
APACHE: Acute Physiology and Chronic Health Evaluation
CCI: Charlson comorbidity index
