# Supplementary figures and images for: A higher prognostic nutritional index is inversely associated with the need for renal replacement therapy in elderly critically Ill surgical patients
Source: BMC Surg. 2025 Oct 21;25:490. doi: 10.1186/s12893-025-03240-w (PMC12538876; doi:10.1186/s12893-025-03240-w)

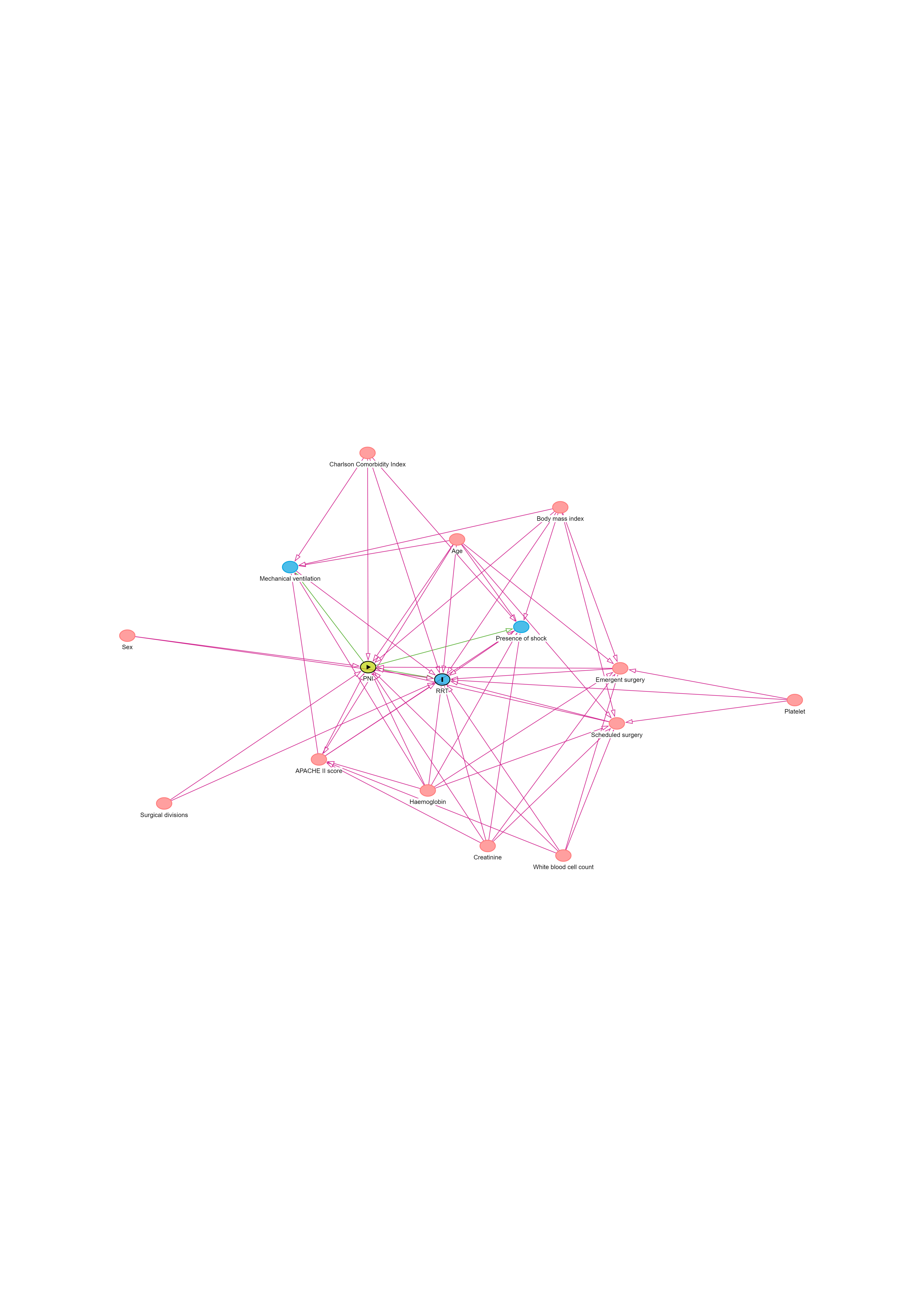

Supplement: Supplementary file 1 — Supplementary Material 1 [file 12893_2025_3240_MOESM1_ESM.png]

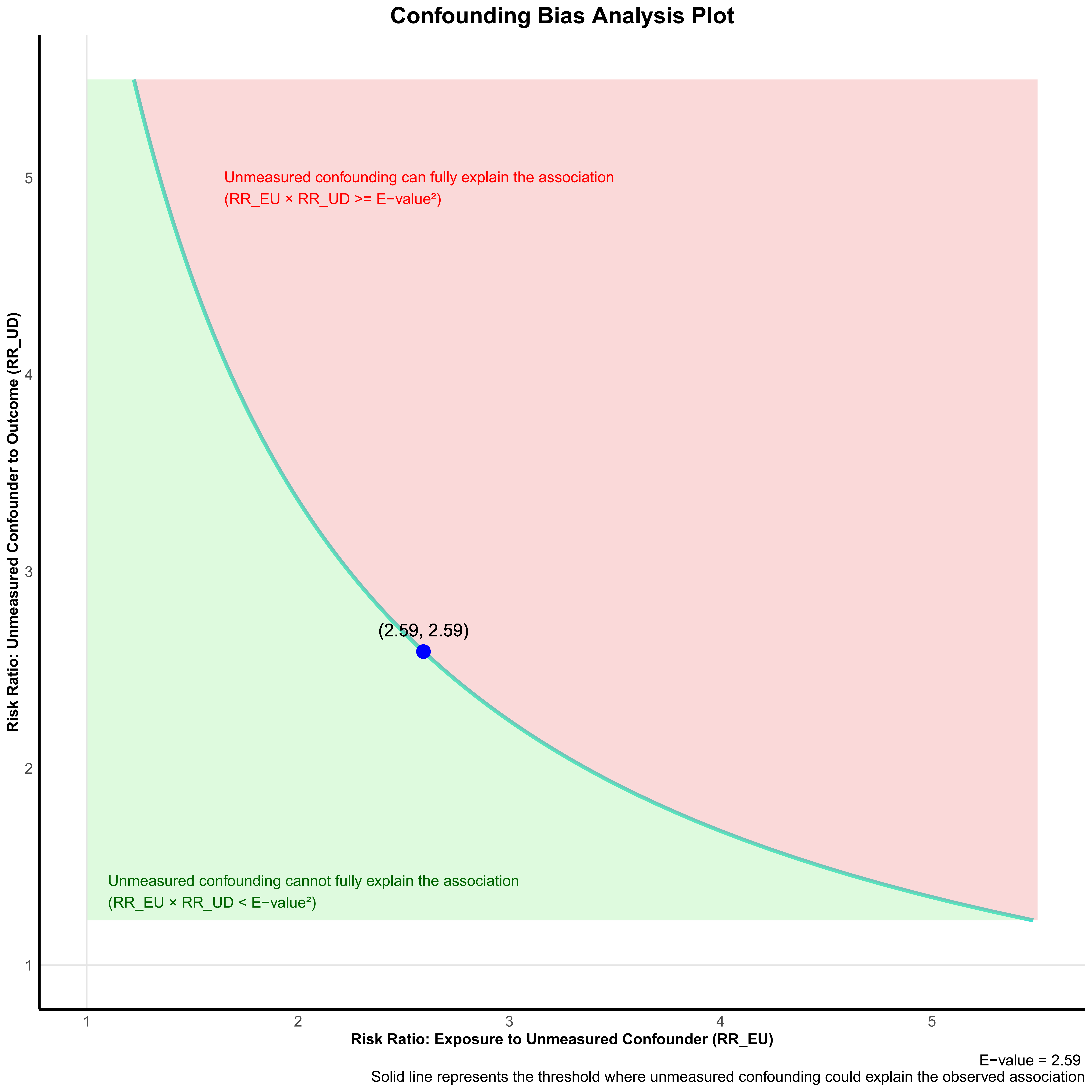

Supplement: Supplementary file 2 — Supplementary Material 2 [file 12893_2025_3240_MOESM2_ESM.png]
