# Supplementary material for: A higher prognostic nutritional index is inversely associated with the need for renal replacement therapy in elderly critically Ill surgical patients
Source: BMC Surg. 2025 Oct 21;25:490. doi: 10.1186/s12893-025-03240-w (PMC12538876; doi:10.1186/s12893-025-03240-w)
Supplement: Supplementary file 3 — Supplementary Material 3 [file 12893_2025_3240_MOESM3_ESM.docx]

**Supplementary Table 1 VIF-based multicollinearity screening**

| variables | VIF values |
| --- | --- |
| PNI | 1.1 |
| Age, years | 1.1 |
| Sex | 1.1 |
| Body mass index (kg/m^2^) | 1.1 |
| Charlson comorbidity index | 1 |
| Surgical divisions | 1.1 |
| Emergent surgery | 1.3 |
| Scheduled surgery | 1.4 |
| APACHE II score | 1.1 |
| Platelet (10^3^/μL) | 1 |
| Creatinine (mg/dL) | 1.2 |
