# Supplementary material for: A higher prognostic nutritional index is inversely associated with the need for renal replacement therapy in elderly critically Ill surgical patients
Source: BMC Surg. 2025 Oct 21;25:490. doi: 10.1186/s12893-025-03240-w (PMC12538876; doi:10.1186/s12893-025-03240-w)
Supplement: Supplementary file 4 — Supplementary Material 4 [file 12893_2025_3240_MOESM4_ESM.docx]

**Supplementary Table 2 Multinomial logistic regression analysis**

|  | NO death and No RRT | Death without RRT | | RRT | |
| --- | --- | --- | --- | --- | --- |
|  | OR (95%CI) | OR (95%CI) | *P*-value | OR (95%CI) | *P*-value |
| (Intercept) | 1.0 (ref.) | 0.1474 (0.0435,0.4989) | 0.0021 | 0.5177 (0.0809, 3.3139) | 0.487 |
| Age, years | 1.0 (ref.) | 1.0340 (1.0223, 1.0458) | <0.0001 | 0.9898 (0.9721, 1.0078) | 0.2653 |
| Sex | 1.0 (ref.) | 1.1671 (0.9695, 1.4048) | 0.1025 | 0.7298 (0.5547, 0.9601) | 0.0244 |
| Body mass index (kg/m^2^) | 1.0 (ref.) | 0.9652 (0.9447, 0.9862) | 0.0013 | 1.0237 (0.9938, 1.0546) | 0.1215 |
| Charlson comorbidity index | 1.0 (ref.) | 1.2451 (1.1785, 1.3156) | <0.0001 | 1.4995 (1.3873, 1.6208) | <0.0001 |
| APACHE II score | 1.0 (ref.) | 1.0512 (1.0375, 1.0651) | <0.0001 | 1.0959 (1.0740, 1.1183) | <0.0001 |
| Platelet count (10^3^/μL) | 1.0 (ref.) | 1.0004 (0.9989, 1.0018) | 0.6164 | 0.9981 (0.9958, 1.0003) | 0.094 |
| Creatinine level (mg/dL) | 1.0 (ref.) | 1.2695 (1.0144, 1.5887) | 0.0371 | 2.6898 (1.9507, 3.7091) | <0.0001 |
| Hemoglobin level (g/dL) | 1.0 (ref.) | 0.9598 (0.9034, 1.0198) | 0.1845 | 0.8794 (0.7984, 0.9686) | 0.0092 |
| Neurosurgical division | 1.0 (ref.) | 0.5699 (0.4057, 0.8005) | 0.0012 | 0.4231 (0.2495, 0.7177) | 0.0014 |
| Cardiovascular surgical division | 1.0 (ref.) | 0.2990 (0.2080, 0.4298) | <0.0001 | 0.7960 (0.4748, 1.3346) | 0.3869 |
| General-colorectal surgery divisions | 1.0 (ref.) | 0.9428 (0.6639, 1.3389) | 0.7421 | 1.2692 (0.7460, 2.1593) | 0.3794 |
| Other surgical division | 1.0 (ref.) | 0.9176 (0.6494, 1.2966) | 0.626 | 1.2109 (0.7256, 2.0210) | 0.4639 |
| Emergency surgery | 1.0 (ref.) | 0.6935 (0.5477, 0.8781) | 0.0024 | 1.1568 (0.7954, 1.6824) | 0.446 |
| Scheduled surgery | 1.0 (ref.) | 0.5331 (0.4364, 0.6513) | <0.0001 | 0.9328 (0.6787, 1.2821) | 0.6683 |
| **PNI (per 10 u)** | **1.0 (ref.)** | **0.7319 (0.6517, 0.8220)** | **<0.0001** | **0.5447 (0.4516, 0.6569)** | **<0.0001** |
